# Did we do everything we could have? Nurses’ contributions to medicines optimization: A mixed‐methods study

**DOI:** 10.1002/nop2.664

**Published:** 2020-10-24

**Authors:** Vera Logan, Sarah Keeley, Kevin Akerman, Elyne De Baetselier, Tinne Dilles, Nia Griffin, Lisa Matthews, Bart Van Rompaey, Sue Jordan

**Affiliations:** ^1^ Department of Nursing Swansea University Swansea United Kingdom; ^2^ Bournemouth University Poole United Kingdom; ^3^ Faculty of Medicine and Health Sciences CRIC (Center of Research and Innovation in Care) NuPhaC University of Antwerp Antwerpen Belgium

**Keywords:** “care gaps”, drug‐related side effects and adverse reactions, interdisciplinary communication, knowledge, nurses, patient safety, pharmaceutical care

## Abstract

**Aim:**

To explore UK professionals’ interpretations of medicines optimization and expansion of nurses’ roles.

**Design:**

This mixed‐methods study sought professionals’ views on nurses’ involvement, competency and engagement in monitoring patients for adverse effects of medicines, monitoring adherence, prescribing and patient education.

**Method:**

An online survey and interviews were undertaken with nurses, doctors and pharmacists in Wales and England, May 2018 to July 2019.

**Results:**

In all, 220 nurses, 17 doctors and 62 pharmacists responded to the online survey, and 24 professionals were interviewed. Nurses were divided over extending their roles, with 123/220 (55.9%) wishing to extend roles in monitoring patients for possible adverse drug reactions (ADRs), 111/220 (50.5%) in adherence monitoring, 121/220 (55.0%) in prescribing and 122/220 (55.4%) in patient education. The best‐qualified nurses were the most willing to increase involvement in monitoring patients for ADRs (aOR 13.00, 1.56–108.01). Interviews revealed that both nurses and doctors assumed the other profession was undertaking this monitoring. Respondents agreed that increasing nurses’ involvement in medicines optimization would improve patient care, but expressed reservations about nurses’ competencies. Collaboration between nurses and doctors was suboptimal (rated 7/10 at best) and between nurses and pharmacists even more so (6/10 at best).

**Conclusion:**

Juxtaposition of datasets identified problems with medicines optimization: although most respondents agreed that increasing nurses’ involvement would positively impact practice, their educational preparation was a barrier. Only ~50% of nurses were willing to expand their roles to fill the hiatus in care identified and ensure that at least one profession was taking responsibility for ADR monitoring.

**Impact:**

To improve multiprofessional team working and promote patient safety, nurse leaders should ensure patients are monitored for possible ADRs by at least one profession. Initiatives expanding nurses’ roles in medicines optimization and prescribing might be best targeted towards the more educated nurses, who have multidisciplinary support.

## 1 INTRODUCTION

The scale and complexity of inadvertent iatrogenic harm from the use and misuse of medicines underlie the World Health Organization's (WHO) Third Global Patient Safety Challenge – to reduce avoidable medication‐related harm by 50% by 2022 (World Health Organisation (WHO), 2017). To address this issue, medicines management, optimization and pharmaceutical care must be prioritized. This study explores professionals’ interpretations of 4 key aspects of medicines management and potential expansion of nurses’ roles in the UK.

## 2 BACKGROUND

Preventable adverse drug reactions and events (ADRs/ ADEs) have proved an intractable problem over the last decade, causing 5%–8% of unplanned hospital admissions (National Institute for Clinical Excellence (NICE) (2015)), rising to ~10%–15% amongst older adults (Oscanoa et al., 2017), costing the UK NHS £1bn‐2.5bn each year. Higher prevalence in larger, prospective studies (Alhawassi et al., 2014) and non‐recognition of ~ 60% events suggest that these figures may be an underestimate (Roulet et al., 2014) and prevalences of 11% and 18% are quoted (Kongkaew et al., 2013; Rydberg et al., 2016). The problem is at least as extensive in developing countries, at ~ 10% of admissions (WHO, 2009), rising to 20% amongst older adults in Africa (Oscanoa et al., 2017).

Most ADEs, ADRs (up to 92%) and medicines’ mismanagement (including errors by patients and professionals) are preventable (NICE, 2015), particularly with additional enhanced monitoring (Gabe et al., 2011; Gandhi et al., 2010). Outside hospital, 15 preventable ADEs occur each 1,000 person‐years, and 25% of these are serious (Gandhi et al., 2010). In hospitals, preventable, mainly dose‐dependent and moderately severe, ADRs affect 3.13 in each 100 patients (95% CI 2.87–3.38, full range 0.006 to 13.3), with lower rates in studies relying on voluntary reporting (Oscanoa et al., 2017; Wolfe et al., 2018).

Medicines optimization (NICE, 2015) is a patient‐focused approach to getting the best from investment in and use of medicines that requires a holistic approach, an enhanced level of patient‐centred professionalism, and partnership between clinical professionals and patients. Medicines optimization and pharmaceutical care require multidisciplinary team working to an extent not previously encountered. Healthcare professionals need to work together to individualize care, monitor outcomes more carefully, review medicines more frequently and support patients (Royal Pharmacological Society (RPS) (2013)). However, nurses’ contributions to medicines optimization and pharmaceutical care remain unexplored.

## 3 THE STUDY

### 3.1 Aims

Building on theories of division of labour (Jordan & Hughes, 2002) and “orphaned tasks” (Jordan, 2002), we report on the readiness of nurses, doctors and pharmacists to engage and optimize four specific responsibilities: monitoring patients for adverse effects of their medicines, adherence to prescribed regimens, prescribing and patient education.

### 3.2 Design

We explored nurses’, doctors’ and pharmacists’ interpretations of nurses’ roles in medicines optimization and pharmaceutical care with a pragmatic mixed‐methods approach (Ford‐Gilboe et al., 1995; Teddlie & Tashakkori, 2012), comprising a cross‐sectional online survey, followed by 24 semi‐structured interviews to investigate actual performance, barriers and facilitators (University of California Los Angeles (UCLA), 2012). We have implemented mixed methods to strengthen the overall study design through triangulation and complementary results of the combined methods (Bryman, 2006). Questions were designed concurrently: neither findings informed the data collection of the other (Jeffries et al., 2019). This paper reports on two UK countries, Wales and England, participating in a pan‐European project (De Baetselier et al., 2020). The good reporting guidelines for mixed‐methods research (National Institutes of Health (NIH). Office of Behavioral and Social Sciences (2018); O'Cathain et al., 2008) were followed (File S1). All questions are reported.

### 3.3 Sample/participation

We contacted the University Health Boards (UHBs), NHS Trusts and private providers in South West Wales and Southern England: the areas where we plan to implement our findings. The questionnaire was distributed through online links to survey as many healthcare professionals as possible. Interviewees were purposively sampled across acute, domiciliary, residential and mental health settings to explore nurses’ roles in practice.

### 3.4 Data collection

In the 10‐min structured questionnaire, all questions were closed, either dichotomous or ordinal polychotomous, with Likert‐type scales, allowing for neutral responses (Nardi, 2018).

For interviews, we sought representative senior NHS practitioners with statistically significant day‐to‐day clinical and managerial responsibility. Private sector participants were sought *via* contact with local nursing home managers and the associated general practitioners (GPs) and pharmacists. In Wales, we recruited from two UHBs and other networks, in England, one Trust and private sector providers were approached. Participant information was shared with professionals who expressed interest. Participants were asked about their perception of the strengths, weaknesses, opportunities and threats of medicines optimization (see File S2 for interview schedule). All interviews were digitally recorded, fully anonymized and professionally transcribed.

### 3.5 Ethical considerations

The survey was approved by the relevant University ethics committees. As we were contacting healthcare professionals, the English NHS Trust required full IRAS (Integrated Research Application System) ethical approval, which was granted on 9 February 2018 (reference number 239960), and cleared by the University on 15 March. When governance checks had been completed (10th April), approval was sought from the R&D (Research and Development) departments in Wales and received on 11 May 2018. IRAS approval was sought for the interview study by application to Health and Care Research Wales and approved on 24 January 2019 (REC reference 19/HCRW/00).

We anticipated minimal physical and emotional risks, as the probability of harm or discomfort was expected to be no higher than ordinarily encountered in participants’ daily lives. There were no questions of a personal or sensitive nature, and no identifying information was sought. Participants were informed of the study's rationale, data collection methods and aims, the voluntary nature of participation, and their rights to withdraw consent without penalty. Informed consent was sought (electronically or in person) and opportunity was given to participants to “phone or email researchers.”

We either did not collect, or immediately deleted, all data relating to names, dates of birth, locations, health, social, racial or ethnic origin, political opinions, religious or philosophical beliefs, or trade union membership. No genetic or biometric data, tissue samples, data concerning health or data concerning a natural person's sex life or sexual orientation were collected (Information Commissioner’s Office (ICO), 2012; Medical Research Council (MRC), 2018).

Throughout data collection and handling, the associated risks of disclosure were mitigated in accordance with General Data Protection Regulations (GDPR) 2018 (MRC, 2018).

### 3.6 Data analysis

All survey variables were described. Ratings were taken as ordinal and compared using Kruskal–Wallis’ independent‐samples tests (Altman, 1991). To explore the predictors of “willingness to extend nurses’ roles” in ADR monitoring, adherence, prescribing and education amongst the 220 nurse respondents, binary outcome variables were obtained by combining categories. The relationship between nurses’ education and willingness to extend their roles was explored using Χ^2^ for trend (Altman, 1991). Binary logistic regression models were constructed using backwards elimination likelihood ratio to select predictor variables. We accounted for sex, education, area of practice, patient population, country and the number of pharmacists and doctors contacted daily. Age and length of experience were tested separately, due to their high collinearity. Data were analysed in the Statistical Package for Social Sciences (SPSS), version 25.0.

Qualitative data were coded and emerging themes identified, as a rolling process (Jordan & Hughes, 1998). Transcripts were analysed with a thematic approach, based on Braun and Clarke (2006), in six stages: familiarization with the data; primary coding of data by applying code labels to the text (see File S3 for examples); identification of themes and patterns; review of themes (with wider research team); detailed analysis and consideration of the relevant themes; and defining outcomes from all data collected. Data analysis and interpretation were discussed by the UK and European teams, and the final analysis reflects joint decisions.

**FIGURE 1:**
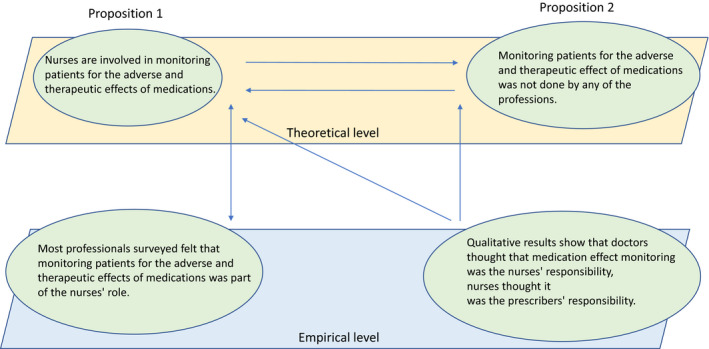
Illustrating the complementary triangulation (adapted from Östlund et al., 2011)

Survey and interview data were integrated around the four responsibilities and the division of labour in these responsibilities, taking a pragmatic perspective of complementary triangulation (Östlund et al., 2011), illustrated in Figure 1. Cross‐cutting themes from the four responsibilities were derived from the data (National Institutes of Health (NIH). Office of Behavioral and Social Sciences, 2018; Foss & Ellefsen, 2002; Denzin, 2012).

### 3.7 Validity and reliability/rigour

The combination of datasets addressing the same question established triangulation of methods (Morse, 2015). Further triangulation across data sources (participants), location (England and Wales) and 2 teams of investigators enhanced transferability. To ensure inter‐rater reliability, stability and validity of our qualitative data analysis, one of the authors who was not actively involved in data collection (SJ) periodically compared data collected by other investigators, checking codes, integrity of the analysis and resolving variances through discussion, in a peer debriefing role (Creswell & Miller, 2000). Purposive sampling of practice areas and congruence of findings with the team's background knowledge of the area enhanced credibility (Polit & Beck, 2012).

The face validity of the survey questions was established by a consortium of nurse researchers and the survey was piloted with 17 nurses, to check its applicability and comparability in different health systems; no changes were needed. No technical problems were reported. The representativeness of the survey sample was checked against externally sourced demographic information. For responses about nurse/doctor collaboration, nurse/pharmacist collaborations, nurses’ competence and team communication, Cronbach's alpha coefficients were: 0.90, 0.93, 0.81 and 0.90, respectively, (1 item for each of the 4 tasks), indicating high internal consistency. Questionnaires that were <50% complete were excluded, as advised by American Association for public opinion research (American Association for Public Opinion Research (AAPOR), 2016).

## 4 RESULTS

### 4.1 Response rate and recruitment

#### 4.1.1 Survey

**FIGURE 2:**
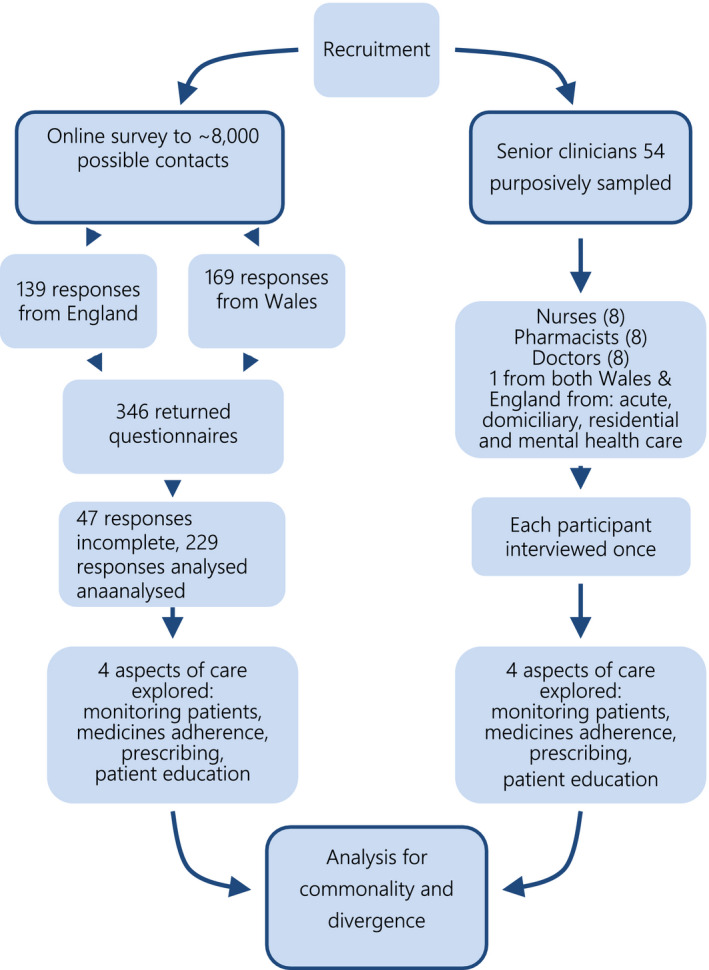
Participant flow diagram

In all, 220 nurses, 17 doctors and 62 pharmacists gave useable responses to the online survey, with primary and secondary care evenly represented. In England, 139 professionals responded from 1,978 possible contacts. In Wales, 169 professionals participated from the ~6,000 in the participating UHB and 78 nurses in other networks. Excluding the 47 questionnaires that were <50% complete left 299 valid responses (179 in Wales and 120 in England; Figure 2).

Demographic details are described in Table S1. A disproportionately high number of nurse respondents held MScs or PhDs (Wales: 29.5% and 9%, England: 19.5% and 3% respectively); 22% (Wales) and 33% (England) of respondents were involved in either research or management.

#### 4.1.2 Interviews

Between February and July 2019, of 14 invitations issued in England, 12 responded positively and 2 failed to respond (both pharmacists). In Wales, 40 professionals were approached: 25 responded positively and 15 negatively or not at all, despite repeated contacts. One respondent subsequently withdrew, unwell, and a substitute was found. No reasons for declining or not responding were given. We had no responses from the Wales PICRIS practices, who are funded to undertake research (National Institute for Social Care and Health Research (NISCHR), 2014).

Interviewees’ experience varied between 30 years (nurse, care home manager) and 3 years (physician – acute care). All interviews were held in private rooms within the participants’ working environments. Interviews lasted between 19 and 31 min.

### 4.2 Monitoring patients for ADRs: “the bit that's missing”

Most nurses surveyed felt that monitoring patients for the adverse and therapeutic effects of medications was part of their role, but a third of doctors and pharmacists disagreed (Table S2a). Interviews revealed a bleaker picture, more congruent with the high proportion of admissions caused by preventable ADRs. Doctors thought nurses were responsible for monitoring patients:

“You prescribe that but you would expect that the nurse would not give it if the blood pressure was really low. Just like with things like painkillers, making sure they wouldn't give it if there were signs of toxicity. … you're putting a lot of trust down to the nurse that's giving the medication that they will make the sensible decision … a very significant amount of responsibility for it.” (England doctor Acute care).

Whereas nurses thought this was the doctors’ responsibility:

“I'm a firm believer that if you prescribe a drug, it is your responsibility to follow up to see whether the outcome is of benefit to the patient. So I think the primary responsibility for a follow up should be with the prescriber. (England, Community nurse), and I think the physicians have to provide information and instruction to the nurses.” (Acute nurse Wales).

A few respondents were aware of this hiatus in care and communications: “*I suppose the bit that's missing is the recording and feeding back part so that any changes can be made.”* (mental health nurse England; Table 1).

**TABLE 1:** THEMATIC ANALYSIS (Quotations selected to illustrate data themes)

Tasks	Themes		
	Unoccupied professional territory, and need for change	Nurse education as a barrier	Workloads and time pressures as a barrier
ADR monitoring	Doctors think nurses monitor: “You prescribe that but you would expect that the nurse would not give it if the blood pressure was really low. Just like with things like painkillers, making sure they wouldn't give it if there were signs of toxicity. … you're putting a lot of trust down to the nurse that's giving the medication that they will make the sensible decision … a very significant amount of responsibility for it.” (England doctor Acute). “If I’ve started a medication I expect them [nurses] to tell me if it has made any difference” (England, doctor, nursing home) Pharmacists felt nurses should be monitoring: “They [nurses] should be looking for adverse effects and making sure the medicines are working for the patients” (England, pharmacist, nursing homes) Nurse thinks doctors monitor: “I'm a firm believer that if you prescribe a drug, it is your responsibility to follow up to see whether the outcome is of benefit to the patient. So I think the primary responsibility for a follow up should be with the prescriber.” (England, Community nurse), “I think the physicians have to provide information and instruction to the nurses. (Acute nurse Wales). And it's not done: “Some of the nurses, they take all the vital signs, the blood pressure, the weight, the MUST scores and they do nothing with it. (…) take those residents with very high blood pressure, and I’m like so what have you done about this, have you let the GP know? Oh no, not yet. And then so why do you bother taking the blood pressure readings if you don't‐‐, if you don't have anything to do with it‐‐, if you're not going to do anything with it?” (England, pharmacist, nursing homes) “Its a fundamentally important job and one that's all too often missed particularly on the health promotion side” (Wales, nurse, mental health) “As doctors we hand it out for hypertension and then we don't hear about the side effects. And then the nurse will say, oh such and such is having problems with her feet, such and such is having problems with her shoes. It's that missing link, you know, there's a side effect, should we be changing our prescription based on those findings? and then you would say who would find those things out? And it would tend to be the nursing staff” (Wales, doctor, acute)	“Nurses may lack knowledge, so that is a weakness ‐ their drug knowledge. And the fact that they should be aware of what they're administering, what they're giving to the patient, that they should have an idea of what it is” (…) Weaknesses of the nurses’ roles in medication management is their knowledge. … nurses aren't as knowledgeable when they're coming into practice. … they're coming into practice having to learn. So whilst I might have learnt and trained many years ago, I felt I came out with a very good basic knowledge. … nurses may lack that knowledge, and they really should be aware of what they're giving to that patient. They should have an idea about what it is. … there's no reason why this can't happen in the university.” (Acute nurse Wales) Results of inadequate education: “I went into a nursing home and they hadn't given Macrogol^®^, which is a laxative, originally on the medication administration record as PRN, so they hadn't given the Macrogol^®^ for 10 days, they just kept writing not required, not required. And then on the 11th day they give an enema. (…) then you see things like loperamide on the MAR chart and you see Movicol^®^ on the MAR chart, “cause maybe the patient had an issue once and they were given loperamide out of hours, they carried on over and over ‐.” (England, pharmacist, Nursing home) “…nebulisers when people are wheezy. The amount of times I've got called and then you just think, okay they're wheezy, they need a nebuliser ‐ but the nurses being able to identify that… an area for improvement.” (England, doctor, acute) “Most times they just give what is on the list and the patient might be suffering from a side effect of the medication. I would say nine times out of ten, they don't pick up that they shouldn't still be giving this medication.” (England, pharmacist, Nursing home)	“I think nurses do struggle sometimes to access ongoing education because they're busy and can't be let off practice.” (England, Pharmacist, community) “I think they would benefit from some kind of annual teaching on medications” (England, pharmacist, mental health). Time pressures in the nursing curriculum: “bigger part of their [nurses] training being based on pharmacology” (England, doctor, acute)
Adherence monitoring	A problem raised in mental health: “I see people coming back to my ward time and time again, very, very unwell. It's very distressing for them and their family, lost years of their lives, and the ongoing burden of increased treatment [high] doses, not to mention the burden of those frequent relapses all because they didn't take their tablets. And did we do everything we could have to prevent that? No we didn't.” (Wales, nurse, mental health)	“I am aware that there are actually standardised approaches to talking to patients to improve their adherence to prescribed medication regimes. But, it's within the competence of any trained nurse to do that work” (England, doctor, mental health)	No comments
Nurse prescribing	Where nurses were working in previously unoccupied professional territory, prescribing was an important component: “if your in a specialist role or you're an advanced nurse practitioner, you know independent prescribing is a real key to your success” (Wales, nurse, acute care) “with independent nurse prescribing you generally have a nurse who's prescribing and been in that set role, or is very mature in practice, or has been in that role for several years so they have lots to add in terms of skillset.” (Wales, nurse, acute) Role confusion centred on overcrowding of professional territory, rather than a gap: “Within prescribing, having several people given free range to prescribe, is the sort of recipe for disaster in general, regardless of who it is” (Wales, nurse, acute) “It could become chaotic and therefore unsafe and we could lose control, is the principal fear and therefore lead to prescribing practices that are unregulated, unsafe and not monitored.” (England, doctor, mental health) ”We lose control of prescribing costs.” (England, doctor, community). “The GPs, when you speak with them, they are furious and like ‐ can you imagine a nurse calling me to give conjunctivitis drops ‐‐, which you can buy over the counter. So the GPs moan, the nurses moan that GPs are not responding fast, and find it ridiculous.” (England, pharmacist, nursing home)	“some of them [nurses] I believe are not very confident with medication” (England, Pharmacist, nursing home) “Sometimes nurse prescribers can get drawn into roles that go beyond their competence. For example, I supervise a nurse prescriber in my community mental health team and one thing I often check are people asking you to do things like provide a diagnosis, provide a risk assessment, because actually if a nurse is asking you to do that you are [supposedly] no more trained to do that than they are.” (England, doctor, mental health)	“Actually having nurse prescribers is potentially the answer [to current lack of medical staff] but that takes them away from their current duties” (England, doctor, mental health) “Nurses are already pretty busy with their own roles at the moment and they may not want to take on an additional role” (Wales, doctor, Community) “actually having enough nurses to cover the essentials before they take on these extended roles” (England, Pharmacist, mental health) “… when you're interrupted constantly, you're trying to write a drug chart, you're trying to re write a drug chart, trying to prescribe, you're in a pressured environment that can obviously happen sometimes, errors can be made” (England, nurse, acute)
Patient education	“when you're giving out medication its making sure that you do tell the patient what they are, what drugs they are taking, often I think we forget to do that so, yeah, making sure, because they [patients] question” (England, nurse, acute) “I think there are a lot of nurses out there who don't go far enough” (Wales, doctor, community)	Nurses lacked the knowledge themselves: “They [nurses] don't seem to have much knowledge about individual drugs when they come out of university so it's probably a steep learning curve for them when they start as a newly qualified nurse.” (England, pharmacist, acute) “Patient education and information varies between individuals and nursing homes, how competent the nurse is on that. Some of them don't even know what the medication is used for so they wouldn't be in a good position to. But all I can say, it varies, it all depends on individual, but the majority I have come across, I wouldn't… (…) Certainly they [nurses] need to be able to know what medicines are for, what side effects they have and give that information when they speak to patients.” (England, pharmacist, nursing home) “If they [patients] ask them [nurses] why they are taking medications they [nurses] should be able to say why” (England, doctor, nursing home)	“In terms of education, again I think nurses should, they have an opportunity, more than doctors, to educate” (Wales, doctor, community)

Note

From 2019, nurse prescribing will be taught in the UK within the preregistration education programme (Nursing and Midwifery Council (NMC) (2018b, 2018a, 2018b, 2018a)). Newly qualified nurses and midwives will be deemed “prescriber ready” and, if working in areas of clinical need, able to access postregistration prescribing programmes, including Community Nurse Prescribing (V150), and Independent Prescribing (V300) within one year of qualification (Nursing and Midwifery Council (NMC) (2018b, 2018a, 2018b, 2018a)).

Nurses gave significantly higher ratings to their collaboration with doctors and pharmacists on monitoring ADRs than did the other professionals (Kruskal–Wallis test, Χ2 22.61, *df* 2, *p* < .001 and Χ2 10.97, *df* 2, *p* .005, respectively). Overall, there was broad agreement that nurses’ involvement would have a positive impact on the quality of patients’ care, and nurses’ involvement in monitoring should be extended. However, interviews revealed an important caveat*:* respondents spontaneously raised nurses’ inadequate preparation in pharmacotherapeutics, congruent with nurses’ assessment of their own competence being significantly higher than that of other healthcare professionals (Χ2 28.69, *df* 2, *p* < .001):

*“Nurses may lack knowledge, so that is a weakness ‐ their drug knowledge. And the fact that they should be aware of what they're administering, what they're giving to the patient, that they should have an idea of what it is.”* (Wales, Acute care nurse).

Many (*n* = 123/220) nurses felt that their roles should be extended to encompass more patient monitoring for ADRs (Table S3a). This willingness was associated with nurses’ education level, particularly doctoral qualification (Table 2).

**TABLE 2:** Medicines optimization roles and nurse education

Nurses’ involvement should be extended in:	What is your highest educational level as a nurse?				Total *n* (%)	Unadjusted analysis		Adjusted analyses			Other statistically significant variables
	Diploma or below *n* (%)	Bachelors *n* (%)	Masters *n* (%)	PhD *n* (%)		*Χ^2^* *df* 1	*p* value	aOR (95% CI) Bachelors	aOR (95% CI) Maters	aOR (95% CI) PhD	
ADR monitoring	22/ 44 (50)	54/ 100 (54)	33/52 (63.5)	13/14 (92.9)	122/210 (58.1)	7.25	0.01	1.18 (0.58–2.41)	1.67 (0.73–3.83)	13.00 (1.56–108.01)	none
Medication adherence	20/ 39 (51.3)	48/94 (51.1)	31/48 (64.6)	11/14 (78.6)	110/195 (56.4)	4.38	0.04	1.13 (0.53–2.44)	1.83 (0.75–4.45)	3.55 (0.84–14.97)	Gender female 0.28 (0.08–1.04)
Prescribing	24/34 (70.6)	52/88 (59.1)	33/46 (71.7)	11/13 (84.6)	120/181 (66.3)	1.21	0.27	0.60 (0.24–1.48)^a^	0.94 (0.32–2.71)	3.10 (0.54–17.59)^a^	Working with: 1–4 pharmacists 2.10 (1.09–4.03): >4 pharmacists 3.14 (0.33–29.58)
Patient education	23/35 (65.7)	51/87(58.6)	35/46 (76.1)	12/13 (92.3)	121/181 (66.9)	4.54	0.03	0.79 (0.35–1.80)	1.72 (0.68–4.65)	6.26 (0.73–54.08)	none

Note

Not all respondents gave their education achievements in the 4 categories analysed and could not be included in these analyses.

Abbreviations: ADR, adverse drug reaction; aOR, adjusted odds ratio; CI, confidence interval; LL, log likelihood.

^a^ Not in final model.

A minority of nurses reported they had never observed ADRs, poor medication adherence or inappropriate prescribing (Table 3). The 14 nurses who had never observed a “side effect” ranged in experience from 0–45 years. Over a third of nurses and doctors did not work with any pharmacists in their daily clinical practice, and around 1 in 7 nurses could not get the help they needed from doctors and pharmacists. A substantial minority of professionals, particularly doctors, did not think that employers’ policies promoted inter‐professional medicines management (Table 4).

**TABLE 3:** Nurses’ experience of medicines monitoring

	Wales *n* (%)	England *n* (%)	Wales *n* (%)	England *n* (%)	Wales *n* (%)	England *n* (%)		Wales *n* (%)	England *n* (%)
	Did you monitor any patients for side effects and therapeutic effects during the last month?		Did you monitor medication adherence for any patients during the last month?		Did you prescribe medication for any patients during last the month?			Did you provide patient education/information about medication use during the last month?	
Yes	89 (69.5)	61 (66.3)	86 (67.2)	52 (56.5)	33 (25.8)	8 (8.7)		94 (73.4)	55 (59.8)
No	34 (26.6)	27 (29.3)	32 (25.0)	26 (28.3)	78 (60.9)	62 (67.4)		17 (13.3)	16 (17.4)
No answer	5 (3.9)	4 (4.3)	10 (7.8)	14 (15.2)	17 (13.3)	22 (23.9)		17 (13.3)	21 (22.8)
If yes, which statements apply?	Last time you observed a side effect, what did you do (more answers possible): *n* (%)		Last time you observed a side effect, what did you do (more answers p*o*ssible): *n* (%)		Last time you observed non‐adherence in a patient, what did you do? (more answers possible): *n* (%)		Questions relating to patient education. If yes, which statements apply?		
I discussed it with a doctor	103 (80.5)	69 (75)	92 (71.8)	57 (62.0)	76 (59.4)	49 (53.3)	Pharmacists, physicians and nurses were well aware of patient education/information provided by each team member	38 (29.7)	20 (21.7)
I discussed it with a pharmacist	35 (27.3)	16 (17.4)	22 (17.2)	14 (15.2)	35 (27.4)	19 (20.6)	I felt qualified to provide patient education or information about medication use	73 (57)	38 (41.3)
I discussed it with a nurse	48 (37.5)	34 (36.9)	38 (29.7)	34 (37.0)	36 (28.1)	24 (26.1)	I received enough information from the doctor to provide patient education or information about medication use	26 (20.3)	17 (18.5)
I discussed it with a patient	67 (52.3)	42 (45.6)	78 (60.9)	48 (52.2)	29 (22.7)	17 (18.5)	I feel other professionals would have given better patient education or information about medication use	20 (15.6)	6 (6.5)
I reported it in the patient file	68 (53.1)	38 (41.3)	68 (53.1)	38 (41.3)	35 (27.3)	15 (16.3)	None of the above answers	4 (3.1)	0
I intervened on my own initiative (e.g. Stopping medication administration)	47 (36.7)	27 (29.4)	24 (18.8)	20 (21.7)	23 (18.0)	8 (8.7)			
I did nothing	0	1 (1.1)	1 (0.8)	0	3 (2.3)	2 (2.2)			
I have never observed a side effect	7 (5.5)	7 (7.6)	9 (7.0)	8 (8.7)	21 (16.4)	14 (15.2)			

**TABLE 4:** The current situation in medicines monitoring

			Agree strongly *n* (%)	Agree *n* (%)	Don't Know *n* (%)	Disagree *n* (%)	Disagree strongly *n* (%)	No response *n* (%)
If I need a doctor to discuss a patient's medicines management/optimization or pharmaceutical care, he/she is easily available for collaboration/ discussion (Question only for nurses)	Wales	Nurses	32 (25)	69 (53.9)	4 (3.1)	13 (10.2)	5 (3.9)	5 (3.9)
	England	Nurses	24 (26.1)	45 (48.9)	5 (5.4)	11 (11.9)	3 (3.3)	4 (4.3)
If I need a pharmacist to discuss a patient's medicines management/ optimization or pharmaceutical care, he/she is easily available for collaboration/ discussion.	Wales	Nurses	31 (24.2)	69 (53.9)	7 (5.5)	13 (10.1)	3 (2.3)	5 (3.9)
	England	Nurses	13 (14.1)	46 (50)	16 (17.4)	6 (6.5)	7 (7.6)	4 (4.3)
My employers’ policies stimulate inter‐professional medicines management.	Wales	Nurses	21 (16.4)	74 (57.8)	14 (10.9)	12 (9.4)	2 (1.6)	5 (3.9)
		Doctors	1 (11.1)	3 (33.3)	3 (33.3)	2 (22.2)	0	0
		Pharmacists	10 (23.8)	25 (59.5)	3 (7.1)	4 (9.5)	0	0
	England	Nurses	10 (10.9)	52 (56.5)	12 (13.0)	13 (14.1)	1 (1.1)	4 (4.3)
		Doctors	0	3 (37.5)	0	3 (37.5)	1 (12.5)	1 (12.5)
		Pharmacists	10 (50)	4 (20)	1 (5)	5 (25)	0	0

### 4.3 Monitoring adherence: “Did we do everything we could?”

Nurses gave significantly higher ratings to their collaboration with doctors on monitoring medication adherence than other professionals (Χ^2^ 19.73, *df* 2, *p* < .001). Their assessment of their own competence was also significantly higher, with pharmacists and doctors in Wales having the most reservations (Χ^2^ 14.90, *df* 2, *p* = .001). Most respondents particularly in mental health, thought more should be done (Table 1, Table S3a):

“…the burden of those frequent relapses all because they didn't take their tablets. And did we do everything we could have to prevent that? No we didn't.” (Wales, nurse, mental health).

Doctors agreed this was a role nurses were able to undertake (Table 1), and involvement in monitoring adherence to medication was viewed positively (Tables S2a and Table S3a). The association between willingness to extend roles in monitoring adherence and nurse education did not reach statistical significance in adjusted analyses (Table 2).

### 4.4 Prescribing: “recipe for disaster” or “potentially the answer”?

Nurses gave higher ratings to their collaboration with doctors on prescribing medication than did the other professionals (Χ2 8.15, *df* 2, *p* = .02). Nurses assessed their own competence to prescribe more highly than did others, reaching borderline statistical significance: the differences were more noticeable in Wales than in England (Χ2 6.07, *df* 2, *p* = .05). Comments included the following: “*sometimes nurse prescribers can get drawn into roles that go beyond their competence* (England, doctor, mental health).

In Wales, 27% of nurses, 78% of doctors and 45% of pharmacists did not consider prescribing medicines to be part of nurses’ roles; in England, it was 31.5%, 62% and 35% (Table S2b). Although most nurses felt that prescribing was a component of their roles, only a minority were active prescribers (26% & 9% in Wales & England). With the exception of doctors in Wales, only a minority stated that extending nurses’ involvement would not be positive. Most nurses thought that their involvement in prescribing should be extended, whilst most doctors and pharmacists thought it should remain unchanged (Table S3b). Concerns related to the practicalities of multiple prescribers, with no‐one in overall control and potential for interactions:

“Within the prescribing, having several people given free range to prescribe I think is the sort of recipe for disaster in general regardless of who it is” (Wales, nurse, acute) and “It could become chaotic and therefore unsafe and that we could lose control, is the principal fear and therefore lead to prescribing practices that are unregulated, unsafe and not monitored.” (England, doctor, mental health).” We lose control of prescribing costs.” (England, doctor, community).

Other reservations concerned existing workloads, and the associated potential for error, whereas support for nurse prescribing centred on specialist roles (Table 1). Willingness to prescribe was predicted by working with pharmacists, rather than education (Table 2).

### 4.5 Patient education: “some [nurses] don't know what the medication is used for”

Nurses gave higher ratings to their collaboration on providing patient education or information about medication use with doctors and pharmacists than did the other professionals (Χ2 8.86, *df* 2, *p* = .01 and Χ2 8.87, *df* 2, *p* = .01). Their assessment of their own competence was also significantly higher (Χ2 20.75, *df* 2, *p* < .001) than experienced by other professionals:

“Patient education and information varies between individuals and nursing homes, how competent the nurse is on that. Some of them don't even know what the medication is used for so they wouldn't be in a good position to. But all I can say, it varies, it all depends on individual, but the majority I have come across, I wouldn't” (England, pharmacist, nursing home).

Most Welsh (80%) and English (62%) nurses thought that providing patient education or information about medication use was a part of nurses’ roles (Table S2b), and 73% of Welsh nurses and 60% of English nurses had provided this in the last month (Table 1). However, only around half (57% and 41%) felt qualified to do this and relied on information from others. Many doctors and pharmacists (44% of doctors and 25% of pharmacists, Table S2b) felt patient education was not a component of nurses’ roles. All professionals thought that the involvement of nurses in providing patient education or information about medication use should be extended (Table S3b):

“In terms of education, again I think nurses should, they have an opportunity more than doctors do to educate” (Wales, doctor, community).

The association between willingness to extend roles in patient education and nurse education did not reach statistical significance in adjusted analyses. (Table 2).

### 4.6 Overarching themes

Complementary triangulation (Östlund et al., 2011) revealed cross‐cutting themes across the 4 tasks examined: care gaps, where no professional is undertaking the task, and each thinks the other does it; inadequate nurses’ education and workload, preventing them from taking on additional roles/education (Table 1).

## 5 DISCUSSION

The nursing workforce is divided regarding role expansion, and a care gap has opened, leaving the monitoring of patients for possible ADRs, non‐adherence and patient education unattended: doctors and nurses each think the other is or should be doing this work, leading to preventable ADRs (Jordan, 2002), sometimes necessitating hospitalization (NICE, 2015; George et al., 2019; Jordan & Hughes, 2019). We identified dissonance in the data: whilst there was consensus that nurses should expand their roles in these domains and tasks, only ~ 50% nurses were willing to do this. Nurses gave significantly higher ratings than other professionals about their own competence and inter‐professional collaborations in monitoring, adherence, prescribing and patient education, corroborated by interviewees’ concerns over nurses’ education and availability.

### 5.1 Care gaps: roles and relationships “It's that missing link”

This study exposed gaps in care: essential tasks unfulfilled by any professionals – through practitioners’ inadvertent misconceptions and absence of structure in policy and management. This could be rectified by expanding roles of nurses (Dilles et al., 2013; Jordan et al., 2015) and pharmacists (RPS, 2013) and mandating structure (Jordan et al., 2019).

Compliance with manufacturers’ therapeutic monitoring recommendations falls short in up to 73% (*n* = 284) patients (Ramia & Zeenny, 2014), and structured nurse‐led patient monitoring addresses this problem (Dijkstra et al., 2018; Dilles et al., 2013; Jones et al., 2016; Jordan, 2002; Jordan et al., 2015, 2019). Interviews indicated that this gap was, in part, attributable to dissonance in expectations: both doctors and nurses thought the other profession was monitoring patients for ADRs, adherence and patient education. Pharmacists and doctors were often aware that this left problems unattended, including administration of anti‐hypertensives to people with hypotension or laxatives to patients with diarrhoea (Table 1; Jordan et al., 2019). Most (90%) nurses agreed that monitoring is a component of nursing roles that would benefit patients (95%), and some 55% of nurses were willing to address this gap by expanding their roles (Jordan et al., 2018).

A similar proportion (55%) of nurses wished to expand their roles to encompass prescribing. Support from pharmacists predicted nurses’ willingness to prescribe (Creedon et al., 2015). Proponents mainly cited prescribing as a component of specialist roles, whereas detractors indicated that risk of errors increases when more than one professional is involved (Assiri et al., 2018), accounting for their reservations.

As the professionals with the most contact with patients, nurses should engage patients in discussions around their medication regularly (Nursing and Midwifery Council (NMC) (2018b, 2018a, 2018b, 2018a); Flanders, 2018, Bowen et al., 2017), but only the more educated nurses were willing to monitor or educate patients. Many nurses (38/149, 26%) did not feel qualified to educate patients about their medicines, and their competence was rated no more highly for patient education than for prescribing.

### 5.2 A workforce divided by education

Although many nurses were willing to expand their roles, some of their colleagues were unconvinced of their competence, particularly in prescribing. Similar discrepancies were reported >40 years ago (Wilson, 1975). Some of these concerns would be allayed by national curricula specifying academic and clinical standards in pharmacology (Jordan et al., 1999) compatible with the needs of service users with multiple comorbidities and associated polypharmacy.

A minority of nurses (6%) had never observed an ADR, despite prevalence of severe preventable events of 0.4% in primary care (Gandhi et al., 2010), 3.13% in secondary care (Oscanoa et al., 2017) and 100% prevalence of medicine‐related problems (Jones et al., 2016; Jordan et al., 2015, 2019). The prevalence of prescribing errors ranges from 2% to 94% of prescriptions (Assiri et al., 2018), so it is surprising that any respondents in the online survey had never seen a prescribing error.

All nurses interviewed and ~50% of nurses responding to the survey felt their roles should be extended to improve medicines optimization, prevent ADRs and minimize iatrogenic harm, but barriers of education and time constraints were apparent. The 2019 changes in the preregistration nursing curriculum may increase nurses’ engagement (Nursing and Midwifery Council (NMC) (2018b, 2018a, 2018b, 2018a)); however, the uptake of current nurse prescribing initiatives is suboptimal (Drennan et al., 2014). Our data indicate that, to succeed, role expansion should be targeted towards better‐educated and motivated nurses: role expansion in medicines optimization may not be for all nurses, and this should be recognized as initiatives are rolled out.

### 5.3 The workforce and the health divide

Expansion of nurses’ roles might ensure timely access to medicines, reduce waiting times and hospital admissions and be a more prudent use of healthcare resources and a positive addition to clinical practice (Health Education England, 2018; Stenner & Courtenay, 2008). However, nurse prescribing depends on support, and strong multidisciplinary relationships (Bowen et al., 2017; Creedon et al., 2015). These are only available in well‐staffed practice areas.

Role expansion has been viewed as the only realistic option to deal with increased demands for health care (Department of Health, 2001), mainly for the old and poor. The creation of a distinct class of non‐specialist healthcare professional to administer medicines to the poor, equivalent to the Russian feldsher, has not been openly considered since the formulation of medical registration in 1858 (Hart, 1988). However, a higher proportion of prescription items are initiated by nurses in areas of socio‐economic deprivation (rho 0.19) and where the number of GPs/ 100,000 population falls below 60 (rho −0.16; Drennan et al., 2014). However, our data indicate that not all nurses are willing to expand their roles, and *caveats* were raised at interview, including the problem of incompatible medicines from multiple prescribers with no clear overall responsibility. To reassure the public, future work should explore whether burgeoning health inequalities might be related to the disproportionate number of nurse prescribing and expanded‐role developments providing services formerly undertaken by doctors in places unattractive to doctors, such as rural areas, former coal‐mining communities and other areas of economic deprivation (Jordan & Griffiths, 2004).

### 5.4 Strengths and limitations

Mixed‐methods research has a long tradition (Hesse‐Biber, 2015), driven, in part, by philosophical pragmatism prioritising solutions to real‐world problems (Dewey, 1938). The synergy achieved by combining narratives and numbers contributes to understanding social phenomena. This work is framed in pragmatic terms (Misak, 2011): we need a reliable solution to the practical problem of medicines optimization in a system characterized by shortages and inequalities. Whilst we acknowledge the risks of dissonance in methodological eclecticism (Teddlie & Tashakkori, 2012), and the risks of subverting either data set (O'Cathain et al., 2008), the convergence and complementarity of findings outweigh the constraints of paradigm dissonance (Denzin, 2012).

Like all self‐reported data, both survey and interview data were vulnerable to social desirability, recall and volunteer biases and respondent error (Campanelli, 2008). Our survey response rate was disappointing, and we attribute this to our method of questionnaire distribution (American Association for Public Opinion Research (AAPOR), 2016; Edwards et al., 2007; McColl et al., 2001), and the delays in obtaining governance approvals. There were low numbers of respondents in some categories, offering only general indications of trends. The age, sex and length of service of survey respondents are in line with the profile of UK nurses (Royal College of Nursing, 2018). However, respondents were self‐selected and might represent the more highly educated and motivated.

We acknowledge the hazards of multiple testing and have limited inferential analyses accordingly; however, as all analyses are indicating the same general trend, we are not basing our interpretations on any single P values (Rothman, 1990). We have no reason to assume that our Trusts/ UHBs are atypical of non‐metropolitan UK, but we make no claim that our respondents represent random samples. We would not wish to make statistical generalizations beyond this sample (Altman, 1991), but the data offer signposts and suggestions as to addressing the “ADR problem” that is causing 5%–8% of unplanned admissions (NICE, 2015), including notes of caution about universal or compulsory expansion of nurses’ roles.

## 6 CONCLUSION

The scale and complexity of inadvertent iatrogenic harm from the use and misuse of prescribed medicines demand change (WHO, 2017) and the closure of the “care gaps” pinpointed here. The solution is seen as effective team working, but workforce and financial constraints suggest this can only be achieved by expanding pharmacists’ and nurses’ roles. However, without structure, patients will continue to be left overmedicated, with preventable ADRs, including hypotension, constipation, sedation, confusion and dyspnoea, as reported here and elsewhere (Jordan et al., 2019).

If iatrogenic harm (and associated admissions) is to be reduced, medicines optimization must be prioritized. Targeting well‐educated nurses for structured interventions to monitor patients offers a practical solution to suboptimal medicines management. To ensure that this reallocation of the division of labour does not further disadvantage areas of socio‐economic deprivation, role expansion must be accompanied by multidisciplinary support and ministry‐level programme changes to reduce ADRs, polypharmacy and miscommunication, as recommended by the WHO (2017).

## CONFLICT OF INTEREST

All authors declare that they have no conflict of interest.

## Supporting information

File S1

File S2

File S3

File S4

## Data Availability

The data that support the findings of this study are available from the corresponding author upon reasonable request.
