# Supplementary material for: Did we do everything we could have? Nurses’ contributions to medicines optimization: A mixed‐methods study
Source: Nurs Open. 2020 Oct 24;8(2):592–606. doi: 10.1002/nop2.664 (PMC7877145; doi:10.1002/nop2.664)
Supplement: Supplementary file 1 — File S1 [file NOP2-8-592-s001.docx]

**Supporting File 1:**

Good Reporting of A Mixed Methods Study (GRAMMS) (O’Cathain et al., 2008)

| Item number | Recommendation | Reported in section |
| --- | --- | --- |
| (1) | Describe the justification for using a mixed methods approach to the research question | 3.2 |
| (2) | Describe the design in terms of the purpose, priority and sequence of methods | 3.2 |
| (3) | Describe each method in terms of sampling, data collection and analysis | 3.3, 3.4, 3.6 |
| (4) | Describe where integration has occurred, how it has occurred and who has participated in it | 3.6, 3.7 |
| (5) | Describe any limitation of one method associated with the present of the other method | 5.4 |
| (6) | Describe any insights gained from mixing or integrating methods | 5.4 |

|  |  |
| --- | --- |
|  |  |
|  |  |
|  |  |
|  |  |
|  |  |
