# Supplementary material for: Did we do everything we could have? Nurses’ contributions to medicines optimization: A mixed‐methods study
Source: Nurs Open. 2020 Oct 24;8(2):592–606. doi: 10.1002/nop2.664 (PMC7877145; doi:10.1002/nop2.664)
Supplement: Supplementary file 2 — File S2 [file NOP2-8-592-s002.docx]

**Supporting File 2: Summary of Interview Schedule**

1. Nurses’ roles in multidisciplinary medicines management / optimisation or pharmaceutical care. Participants were shown a list of four responsibilities:
   1. monitoring & following-up adverse / therapeutic effects,
   2. monitoring & following-up medicines adherence,
   3. decision making on medicines use, including prescribing medicines, excluding preparation / administration, and
   4. providing patient education and information about medicines and asked what should be changed, added or removed.
2. Within these 4 responsibilities, what tasks should nurses perform?
3. What would ideal collaboration and communication between nurses and other professionals look like in a-d above?
4. To optimise care, what are the strengths, weaknesses, opportunities and threats? Reference the responsibilities a-d above.
