# Supplementary material for: Did we do everything we could have? Nurses’ contributions to medicines optimization: A mixed‐methods study
Source: Nurs Open. 2020 Oct 24;8(2):592–606. doi: 10.1002/nop2.664 (PMC7877145; doi:10.1002/nop2.664)
Supplement: Supplementary file 3 — File S3 [file NOP2-8-592-s003.docx]

**Supporting File 3: Coded extracts of interviews**

| Theme | Code | **Acute Pharmacist - Wales** |
| --- | --- | --- |
| Care gap  Education as a barrier  Care Gap  Education as a barrier | Nurses and patient monitoring  Education  Prescribing  Lack of knowledge prevents nurses contributing more.  Nursing role should focus on the quality aspect of medicines management.  Prescribing  Lack of knowledge leads to inability to counsel patients effectively. | I think they [nurses] should be focusing on the quality aspects, rather than the kind of the physical act of administering medicine. ...expertise around the monitoring and ensuring the drug is safe to give now before giving, *I would like them to be* concentrating on that and you use other kind of maybe professions or maybe--, maybe non-registrants who are supervised by a nurse to give the medicines.  Any problems that you identify, any discrepancies, you highlight with the prescriber or resolve them if it’s within your capabilities. So just to get nurses a bit more involved in that, so say it hasn’t received medicines reconciliation by someone and a nurse is capable of doing that before going ahead and giving the medicines.....  on the whole nurses are probably not trained to do it, *they probably don’t get the training to do it.*  So prescribing, yeah, definitely, you know, more involvement in that would be good,  It’s knowledge. Because there is a ceiling to the knowledge. There is support out there but I suppose if the knowledge was greater they could do more. However, I think you’ve got to be realistic here, they have to know a lot of other things that other--, like, say, a pharmacist or a doctor doesn’t know, so that’s where the whole multidisciplinary team comes in.  You *want* them focused on patient quality aspect, so counselling, ensuring the medicine’s safe to give, doing the appropriate monitoring and responding to the appropriate monitoring, that kind of stuff. So you want someone else chasing the medicines,  If a nurse knows the medicine why can’t he or she be allowed to prescribe it all? So giving patients access to medicines that nurses think they should have I think is reasonable, you know, so that--, should all nurses be prescribers? Possibly, yeah. Possibly as part of the--, if it’s in their remit and then, you know. Yeah, so I think all nurses--, it shouldn’t be just a specialist role to be a prescriber, all nurses should be prescribers maybe, and then you use those skills according  Going back to your weaknesses with nurses, I think counselling is possibly one, having that knowledge to be able to effectively counsel a patient about all the drugs. It’s a difficult one because how do you maintain that knowledge since you may not be using it all the time, but I think a bit more of that would be useful. |
|  |  | Community pharmacist England |
| Care Gap | Division of labour  Care gap  Suboptimal  Care gap  Nurses’ roles /  Division of Labour | So monitoring, following up, adverse… If you’re a prescriber that’s definitely part of your job You need to discuss with the patient what monitoring is required and you need to make sure it’s been done, If you’re not doing it yourself, then it’s been done and that would apply to a nurse prescriber. All health care professionals have a responsibility around adverse effects in terms of reporting if appropriate. So you’re thinking sort of yellow card scheme and things like that. - actually the monitoring following up of medicines are absolutely--, those two really can be sort of wrapped up into what I would say is good medication review. There’s *an awful lot of medication review done that isn’t good because it doesn’t involve the patient*, so I think we understand that now particularly patients on lots of medicines that actually, you know, there is risk benefits change over time. So actually this idea of sort of *fire and forget is wrong really*, so yeah I think if the nurse is either prescribing it or responsible. They could be not a prescriber but responsible through diabetes clinic for actually doing a lot of that on behalf of the GP. Decision making on prescribed medicines excluding preparation and administration: they’re [nurses are] certainly making lots of decisions on medicines used, even if they’re a prescriber or not. |
|  |  | Community nurse England |
| Education as a barrier | Education and ability  Division of Labour  Nurses’ roles  Division of Labour uncertain  Care Gap | If you’re going to be administering medication, you must be trained in that and you must keep up to date in terms of your knowing what to do, who to contact, how to escalate the patient and your concerns. How to record detailed information: we have a yellow card scheme where we can highlight if we think it’s a particular drug. Anybody can do that, it doesn’t necessarily have to be a prescriber. So I think they need more awareness over that. (…) So they may well be actually quite well placed to manage that in the great scheme of things of priorities. So yes, it must be part of their responsibility.  I'm a firm believer that if you prescribe a drug, it is your responsibility to follow up to see whether the outcome is of benefit to the patient. So I think the primary responsibility for a follow up should be with the prescriber. However, I think nurses are, again because they’re often seeing the patient at different times, are perhaps a little bit more well placed to pick up on things that might add to that follow up review. And I don’t think it should be solely their responsibility. |
