# Supplementary material for: Did we do everything we could have? Nurses’ contributions to medicines optimization: A mixed‐methods study
Source: Nurs Open. 2020 Oct 24;8(2):592–606. doi: 10.1002/nop2.664 (PMC7877145; doi:10.1002/nop2.664)
Supplement: Supplementary file 4 — File S4 [file NOP2-8-592-s004.docx]

Supporting Information file 4: additional tables

Table 1. Demographics and employment details of respondents

| Profession | | Nurses | | Doctors | | Pharmacists | |
| --- | --- | --- | --- | --- | --- | --- | --- |
| Country | | Wales | England | Wales | England | Wales | England |
| Respondents | | 128 | 92 | 9 | 8 | 42 | 20 |
| Sex | Women n (%) | 117 (91.4) | 86 (93.5) | 6 (66.7) | 6 (75) | 28 (66.7) | 14 (70) |
|  | Men n (%) | 11 (8.6) | 6 (6.5) | 3 (33.3) | 2 (25) | 14 (33.3) | 6 (30) |
| Age | Mean [SD] | 45.9  [9.6] | 42.1 [10.8] | 36.3 [9.6] | 39.2 [15.8] | 37.4  [10.5] | 37.3 [11.5] |
| Years of experience | Mean [SD] | 21.8  [10.9] | 18.9 [11.9] | 11.2 [9.3] | 11.9 [17.1] | 14.9  [10.9] | 13.1 [11.5] |
| Highest educational level (question only for nurses) | PhD n(%) | 11 (8.6) | 3 (3.3) |  |  |  |  |
|  | MSc / MN n(%) | 35 (27.3) | 17 (18.5) |  |  |  |  |
|  | BSc /BN n(%) | 57 (44.5) | 43 (46.7) |  |  |  |  |
|  | Diploma n(%) | 20 (15.6) | 24 (26.1) |  |  |  |  |
|  | Other n(%) | 5 (3.9) | 5 (5.4) |  |  |  |  |
| Days/ year in non-mandatory education | >2 n(%) | 105 (82.0) | 71 (77.2) | 7 (77.8) | 7 (87.5) | 32 (76.2) | 16 (80) |
|  | 1-2 n(%) | 13 (10.2) | 15 (16.3) | 0 | 0 | 7 (16.7) | 2 (10) |
|  | <1 n(%) | 4 (3.1) | 2 (2.2) | 0 | 0 | 2 (4.8) | 2 (10) |
|  | 0 n(%) | 1 (0.8) | 0 | 2 (22.2) | 0 | 1 (2.4) | 0 |
|  | No response | 5 (3.9) | 4 (4.3) | 0 | 1(12.5) | 0 | 0 |
| Medications management education since qualifying | Yes n(%) | 82 (64.1) | 45 (48.9) | Question not for doctors or pharmacists | | | |
|  | No n(%) | 40 (31.5) | 43 (46.7) |  |  |  |  |
|  | No response n(%) | 6 (4.7) | 4 (4.3) |  |  |  |  |
| Practice area  (more than one answers possible) | Community n(%) | 56 (43.8) | 19 (20.7) | 2 (22.2) | 1 (12.5) | 17 (40.5) | 3 (15) |
|  | Residential care n(%) | 1 (0.8) | 4 (4.3) | 0 | 0 | 0 | 0 |
|  | Hospital n(%) | 59 (46.1) | 61 (66.3) | 6 (66.7) | 5 (62.5) | 21 (50) | 16 (80) |
|  | Other n(%) | 12 (9.4) | 8 (8.7) | 1 (11.1) | 2 (25) | 4 (9.5) | 1 (5) |
|  | No response n(%) | 0 | 0 | 0 | 0 | 0 | 0 |
| Patient population | 0-17 years of age | 12 (9.4) | 4 (4.3) | 0 | 0 | Question not for pharmacists | |
|  | 18-64 years n(%) | 26 (20.3) | 22 (23.9) | 1 (11.1) | 3 (37.5) |  |  |
|  | >64 years n(%) | 12 (9.4) | 18 (19.6) | 1 (11.1) | 0 |  |  |
|  | >one group n(%) | 73 (57) | 44 (47.8) | 7 (77.8) | 4 (50) |  |  |
|  | No response n(%) | 5 (3.9) | 4 (4.3) | 0 | 1 (12.5) |  |  |
| Clinical domain | Patient care n(%) | 101 (77.7) | 71 (66.3) | 7 (70) | 7 (100) | 35 (68.6) | 15 (60) |
|  | Research n(%) | 8 (6.2) | 21 (19.6) | 2 (20) | 0 | 3 (5.8) | 2 (8) |
|  | Management n(%) | 21 (16.1) | 15 (14) | 1 (10) | 0 | 13 (25.4) | 8 (32) |
| Hours/week in clinical practice | Mean [SD] | 31.8  [9.7] | 30.7 [13.8] | 36.0 [16.1] | 41.7 [7.5] | 29.3  [10.8] | 30.4 [12.8] |
| How many nurses do you work with daily? | None n(%) | 1 (0.8) | 5 (5.4) | 0 | 0 | 7 (16.7) | 2 (10) |
|  | 1-4 n(%) | 31 (24.2) | 30 (32.6) | 5 (55.5) | 2 (25) | 15 (35.7) | 6 (30) |
|  | 5-10 n(%) | 38 (29.7) | 23 (25) | 1 (11.1) | 1 (12.5) | 13 (30.9) | 5 (25) |
|  | 10+ n(%) | 51 (39.8) | 29 (31.5) | 2 (22.2) | 4 (50) | 7 (16.7) | 7 (35) |
|  | No response n(%) | 7 (5.5) | 5 (5.4) | 1 (11.1) | 1 (12.5) | 0 | 0 |
| How many doctors do you work with daily? | None n(%) | 8 (6.3) | 6 (6.5) | 0 | 0 | 5 (11.9) | 2 (10) |
|  | 1-4 n(%) | 58 (45.3) | 32 (34.8) | 3 (33.3) | 2 (25) | 17 (40.4) | 8 (40) |
|  | 5-10 n(%) | 35 (27.3) | 26 (28.3) | 3 (33.3) | 3 (37.5) | 14 (33.3) | 7 (35) |
|  | 10+ n(%) | 17 (13.3) | 22 (23.9) | 2 (22.2) | 2 (25) | 6 (14.2) | 3 (15) |
|  | No response n(%) | 10 (7.8) | 6 (6.5) | 1 (11.1) | 1 (12.5) | 0 | 0 |
| How many pharmacists do you work with daily? | None n(%) | 38 (29.7) | 37 (40.2) | 3 (33.3) | 6 (75) | 12 (28.5) | 1 (5) |
|  | 1-4 n(%) | 77 (60.2) | 43 (46.7) | 4 (44.4) | 1 (12.5) | 16 (38) | 5 (25) |
|  | 5-10 n(%) | 1 (0.8) | 3 (3.3) | 0 | 0 | 6 (14.2) | 6 (30) |
|  | 10+ n(%) | 2 (1.6) | 2 (2.2) | 1 (11.1) | 0 | 8 (19) | 8 (40) |
|  | No response n(%) | 10 (7.8) | 10 (10.9) | 1 (11.1) | 1 (12.5) | 0 | 0 |

Table 2a: Nursing role in relation to monitoring ADRs and adherence

|  | | | Strongly agree | Agree | Don’t know | Disagree | Strongly disagree | Not applicable | No response |
| --- | --- | --- | --- | --- | --- | --- | --- | --- | --- |
| In your daily clinical practice, monitoring side effects and therapeutic effects of medication is a part of nurses’ roles. | Wales | Nurses | 61 (47.7) | 54 42.2) | 0 | 6 (4.7) | 0 | 2 (1.6) | 5 (3.9) |
|  |  | Doctors | 2 (22.2) | 4 (44.4) | 0 | 1 (11.1) | 2 (22.2) | 0 | 0 |
|  |  | Pharmacists | 1 (2.4) | 18 (42.9) | 1 (2.4) | 10 (23.8) | 4 (9.5) | 8 (19) | 0 |
|  | England | Nurses | 41 (44.6) | 36 (39.1) | 3 (3.4) | 5 (5.7) | 1 (1.1) | 2 (2.3) | 4 (4.3) |
|  |  | Doctors | 0 | 3 (37.5) | 0 | 3 (37.5) | 1 (12.5) | 0 | 1 (12.5) |
|  |  | Pharmacists | 3 (15) | 10 (50) | 1 (5) | 3 (15) | 1 (5) | 0 | 2 (10) |
| Nurses involved in monitoring side effects and therapeutic effects has/would have a positive impact on the quality of patient care an medicines management. | Wales | Nurses | 81 (63.3) | 40 (31.3) | 1 (0.8) | 1 (0.8) | 0 | 0 | 5 (3.9) |
|  |  | Doctors | 4 (44.4) | 5 (55.5) | 0 | 0 | 0 | 0 | 0 |
|  |  | Pharmacists | 11 (26.2) | 25 (59.5) | 2 (4.8) | 4 (9.5) | 0 | 0 | 0 |
|  | England | Nurses | 45 (48.9) | 35 (38) | 7 (7.6) | 1 (1.1) | 0 | 0 | 4 (4.3) |
|  |  | Doctors | 3 (37.5) | 3 (37.5) | 1 (12.5) | 0 | 0 | 0 | 1 (14.3) |
|  |  | Pharmacists | 8 (40) | 12 (60) | 0 | 0 | 0 | 0 | 0 |

| In your daily practice, monitoring medication adherence is a part of nurses’ roles. n(%) | Wales | Nurses | 57 (44.5) | 48 (37.5) | 0 | 1 (0.8) | 0 | 12 (9.4) | 10 (7.8) |
| --- | --- | --- | --- | --- | --- | --- | --- | --- | --- |
|  |  | Doctors | 4 (44.4) | 2 (22.2) | 0 | 1 (11.1) | 0 | 0 | 2 (22.2) |
|  |  | Pharmacists | 4 (9.5) | 23 (54.8) | 1 (2.4) | 6 (14.3) | 1 (2.4) | 3 (7.1) | 4 (9.5) |
|  | England | Nurses | 40 (43.5) | 27 (29.3) | 2 (2.2) | 5 (5.4) | 1 (1.1) | 3 (3.3) | 14 (15.2) |
|  |  | Doctors | 2 (25) | 3 (37.5) | 0 | 0 | 1 (12.5) | 1 (12.5) | 1 (12.5) |
|  |  | Pharmacists | 5 (25) | 7 (35) | 2 (10) | 6 (30) | 0 | 0 | 0 |
| Nurses involved in monitoring medication adherence has/would have a positive impact on the quality of medicines management. n(%) | Wales | Nurses | 71 (55.5) | 44 (34.3) | 3 (2.3) | 0 | 0 | 0 | 10 (7.8) |
|  |  | Doctors | 4 (44.4) | 4 (44.4) | 0 | 0 | 0 | 0 | 1 (11.1) |
|  |  | Pharmacists | 9 (21.4) | 25 (59.5) | 2 (4.8) | 2 (4.8) | 0 | 0 | 4 (9.6) |
|  | England | Nurses | 41 (44.6) | 33 (35.9) | 0 | 1 (1.1) | 0 | 0 | 17 (18.5) |
|  |  | Doctors | 3 (37.5) | 3 (37.5) | 1 (12.5) | 0 | 0 | 0 | 1 (12.5) |
|  |  | Pharmacists | 11 (55) | 8 (40) | 0 | 1 (5) | 0 | 0 | 0 |

Table 2b: Nursing role in relation to prescribing medication and providing patient education/information about medication use

|  | | | Strongly agree | Agree | Don’t Know | Disagree | Strongly disagree | Not applicable | No response |
| --- | --- | --- | --- | --- | --- | --- | --- | --- | --- |
| In your daily practice, prescribing medication is a part of nurses’ roles. n(%) | Wales | Nurses | 20 (15.6) | 34 (26.6) | 1 (0.8) | 19 (14.8) | 15 (11.7) | 23 (18.0) | 16 (12.5) |
|  |  | Doctors | 0 | 1 (11.1) | 0 | 3 (33.3) | 4 (44.4) | 0 | 1 (11.1) |
|  |  | Pharmacists | 1 (2.4) | 9 (21.4) | 0 | 16 (38.1) | 3 (7.1) | 4 (9.5) | 9 (21.4) |
|  | England | Nuses | 8 (8.7) | 14 (15.2) | 0 | 17 (18.5) | 12 (13.0) | 19 (20.7) | 22 (23.9) |
|  |  | Doctors | 2 (25.0) | 0 | 0 | 3 (37.5) | 2 (25) | 0 | 1 (12.5) |
|  |  | Pharmacists | 3 (15.0) | 6 (30.0) | 0 | 6 (30.0) | 1 (5.0) | 3 (15.0) | 1 (5.0) |
| More nurses involved in prescribing medication has/would have a positive impact on the quality of care and medicines optimisation. n(%) | Wales | Nurses | 38 (29.7) | 56 (43.8) | 9 (7.0) | 6 (4.7) | 3 (2.3) | 0 | 16 (12.5) |
|  |  | Doctors | 1 (11.1) | 1 (11.1) | 0 | 5 (55.5) | 1 (11.1) | 0 | 1 (11.1) |
|  |  | Pharmacists | 4 (9.5) | 15 (35.7) | 3 (7.1) | 10 (23.8) | 1 (2.4) | 0 | 9 (21.4) |
|  | England | Nurses | 14 (15.2) | 32 (34.8) | 11 (12.0) | 13 (14.1) | 0 | 0 | 22 (23.9) |
|  |  | Doctors | 2 (25.0) | 2 (25.0) | 2 (25.0) | 1 (12.5) | 0 | 0 | 1 (12.5) |
|  |  | Pharmacists | 4 (20.0) | 9 (45.0) | 2 (10.0) | 3 (15.0) | 1 (5.0) | 0 | 1 (5.0) |
| In your daily practice, providing patient education or information about medication use is a part of nurses’ roles n(%) | Wales | nurses | 65 (50.8) | 38 (29.7) | 0 | 4 (3.1) | 0 | 4 (3.1) | 17 (13.3) |
|  |  | doctors | 3 (33.3) | 3 (33.3) | 0 | 1 (11.1) | 1 (11.1) | 0 | 1 (11.1) |
|  |  | pharmacists | 1 (2.4) | 21 (50) | 1 (2.4) | 8 (19.0) | 0 | 1 (2.4) | 10 (23.8) |
|  | England | nurses | 27 (29.3) | 30 (32.6) | 2 (2.2) | 9 (9.8) | 0 | 3 (3.3) | 21 (22.8) |
|  |  | doctors | 0 | 3 (37.5) | 0 | 3 (37.5) | 1 (12.5) | 0 | 1 (12.5) |
|  |  | pharmacists | 3 (15) | 8 (40) | 0 | 5 (25) | 1 (5) | 1 (5) | 2 (10) |
| Nurses involved in providing more patient education or information about medication use have/would have a positive impact on the quality of medicines management n(%) | Wales | nurses | 70 (54.7) | 37 (28.9) | 3 (2.3) | 1 (0.8) | 0 | N/A | 17 (13.3) |
|  |  | doctors | 4 (44.4) | 3 (33.3) | 0 | 1 (11.1) | 0 | N/A | 1 (11.1) |
|  |  | pharmacists | 7 (16.7) | 21 (50) | 2 (4.8) | 1 (2.4) | 1 (2.4) | N/A | 10 (23.8) |
|  | England | nurses | 29 (31.5) | 38 (41.3) | 2 (2.2) | 1 (1.1) | 1 (1.1) | N/A | 21 (22.8) |
|  |  | doctors | 2 (25) | 5 (62.5) | 0 | 0 | 0 | N/A | 1 (12.5) |
|  |  | pharmacists | 5 (25) | 9 (45) | 1 (5) | 3 (15) | 0 | N/A | 2 (10) |
|  |  |  |  |  |  |  |  |  |  |

Table 3a: Extension of nursing roles and current quality of inter-professional collaboration in relation to monitoring ADRs and adherence

|  | | Nurses | | Doctors | | Pharmacists | |
| --- | --- | --- | --- | --- | --- | --- | --- |
|  |  | Wales | England | Wales | England | Wales | England |
| The involvement of nurses in monitoring side effects and therapeutic effects should be: | Extended | 72 (56.3) | 51 (55.4) | 6 (66.7) | 5 (62.5) | 23 (54.8) | 14 (70) |
|  | Remain the same | 50 (39.1) | 35 (38.0) | 3 (33.3) | 1 (12.5) | 15 (35.7) | 6 (30) |
|  | Restricted | 1 (0.8) | 2 (2.2) | 0 | 0 | 3 (7.1) | 0 |
|  | No answer | 5 (3.9) | 4 (4.3) | 0 | 2 (25) | 1 (2.4) | 0 |
| From your current experience, please rate the following items with a score of 0 – 10 (0 = low quality, 10 = high quality) on the current quality of …  Median [25^th^-75^th^ centiles], (full range) | Collaboration between nurses and doctors on monitoring side effects and therapeutic effects: | 7.0  [5.0–8.0]  (0 – 10) | 7.0  [5.0–8.5]  (0 – 10) | 5.5  [4.3–8.0]  (2 – 8) | 2.0  [1.5–8.0]  ( 1 – 9) | 5.0  [3.8–7.0]  (2 – 10) | 5.0  [4.0–8.0]  (2 – 10) |
|  | Collaboration between nurses and pharmacists on monitoring side effects and therapeutic effects: | 6.0  [5.0–8.0]  (0 – 10) | 5.0  [2.0–7.5]  (0 – 10) | 1.5  [0.0–5.8]  (0 – 8) | 2.0  [1.0–2.0]  (1 – 2) | 5.5  [5.0–8.0]  (2 – 10) | 5.0  [4.0–6.0]  (0 – 9) |
|  | Nurses’ competence in monitoring side effects and therapeutic effects: | 7.0  [5.0–8.0]  (0 – 10) | 6.0  [5.0–8.0]  (2 – 10) | 5.5  [4.3–6.3]  (2 – 7) | 2.0  [1.0–8.0]  (1 – 8) | 5.0  [3.8–7.0]  (1 – 8) | 5.0  [3.0–8.0]  (2 – 10) |
|  | Interprofessional communication on monitoring side effects and therapeutic effects: | 7.0  [5.0–8.0]  (0 – 10) | 6.0  [4.0–7.0]  (1 – 10) | 6.0  [3.8–7.0]  (3 – 7) | 3.0  [1.5–5.5]  (1 – 8) | 5.0  [4.0–7.0]  (1 – 10) | 5.0  [3.0–7.0]  (2 – 10) |

| The involvement of nurses in monitoring medication adherence should be: | Extended | 66 (51.6) | 45 (48.9) | 6 (66.7) | 3 (37.5) | 20 (47.6) | 14 (70) |
| --- | --- | --- | --- | --- | --- | --- | --- |
|  | Remain the same | 52 (40.6) | 32 (34.8) | 2 (22.2) | 4 (50.0) | 17 (40.5) | 6 (30) |
|  | Restricted | 0 | 1 (1.1) | 0 | 0 | 1 (2.4) | 0 |
|  | No response | 10 (7.8) | 14 (15.2) | 2 (22.2) | 1 (12.5) | 4 (9.5) | 0 |
| From your current experience, rate the following items with a score of 0 – 10 (0 = low quality, 10 = high quality) on the current quality of:  Median [25^th^-75^th^ centiles], (full range) | Collaboration between nurses and doctors on monitoring medication adherence Median [25^th^-75^th^ centiles], (full range) | 7.0  [5.0–8.0]  (0 - 10) | 7.0  [5.0–8.0]  (0 - 10) | 5.5  [4.5-7.3]  (3 - 8) | 6.0  [4.0-8.5]  (3 - 9) | 5.0  [3.8–7.3]  (2 – 10) | 5.0  [2.0–7.0]  (0 – 10) |
|  | Collaboration between nurses and pharmacists on monitoring medication adherence | 6.0  [4.0–8.0]  (0 – 10) | 5.0  [2.0–6.5]  (0 – 10) | 2.5  [0.8–5.8]  (0 – 8) | 3.0  [2.5–7.5]  (2 – 9) | 7.0  [4.8–8.0]  (2 – 10) | 5.0  [3.0–6.0]  (0 – 9) |
|  | Nurses’ competence in monitoring medication adherence | 7.5  [6.0–8.0]  (0 – 10) | 7.0  [5.0–8.0]  (1 – 10) | 5.0  [3.8–7.0]  (3 – 7) | 7.0  [3.5–9.0]  (3 – 9) | 5.0  [4.8–7.3]  (1 – 10) | 5.0  [2.0–8.0]  (0 – 10) |
|  | Interprofessional communication on monitoring medication adherence | 7.0  [5.0–8.0]  (0 – 10) | 6.0  [4.5–8.0]  (0 – 10) | 4.5  [2.8–6.3]  (2 – 7) | 5.0  [2.0–7.5]  (1 – 8) | 5.5  [5.0–7.3]  (1 – 10) | 5.0  [2.0–7.0]  (0 – 10) |

Table 3b: Extension of nursing roles and current quality of inter-professional collaboration in relation to prescribing medication and providing patient education/information about medication use

|  | | Nurses | | Doctors | | Pharmacists | |
| --- | --- | --- | --- | --- | --- | --- | --- |
|  |  | Wales | England | Wales | England | Wales | England |
| The involvement of nurses in prescribing medication should be: | Extended | 79 (61.7) | 42 (45.7) | 2 (22.2) | 3 (37.5) | 13 (31.0) | 10 (50.0) |
|  | Remain the same | 26 (20.3) | 23 (25.0) | 4 (44.4) | 3 (37.5) | 16 (38.1) | 7 (35.0) |
|  | Restricted | 7 (5.5) | 5 (5.4) | 2 (22.2) | 1 (12.5) | 4 (9.5) | 2 (10.0) |
|  | No response | 16 (12.5) | 22 (23.9) | 1 (11.1) | 1 (12.5) | 9 (21.4) | 1 (5.0) |
| From your current experience, please rate the following items with a score of 0 – 10 (0 = low quality, 10 = high quality) on the current quality of…  Median [25^th^-75^th^ centiles], (full range) | Collaboration between nurses and doctors on prescribing medication | 7.0  [5.0-8.0]  (0 – 10) | 7.0  [4.0-8.0]  (0 – 10) | 3.0  [3.0-5.0]  (0 – 9) | 5.0  [5.0-8.0]  (2 – 9) | 5.0  [4.0-7.0]  (2 – 10) | 7.0  [5.0-8.0]  (3 – 10) |
|  | Collaboration between nurses and pharmacists on prescribing medication | 6.5  [5.0-8.0]  (0 – 10) | 5.0  [2.0-8.0]  (0 – 10) | 1.0  [0.0-6.0]  (0 – 8) | 5.0  [2.0-5.0]  (2 – 8) | 5.0  [4.0-7.0]  (0-10) | 7.0  [5.0-7.0]  (3 – 9) |
|  | Nurses’ competence in prescribing medication | 7.0  [5.0-8.0]  (0 – 10) | 5.0  [3.0-7.0]  (0 – 10) | 3.0  [3.0-5.0]  (0 – 5) | 5.0  [3.0-9.0]  (1 – 10) | 5.0  [4.0-6.0]  (1 – 10) | 6.0  [4.5-8.5]  (2 – 10) |
|  | Interprofessional communication on prescribing medication | 7.0  [5.0-8.0]  (0 – 10) | 6.0  [3.0-8.0]  (0 – 10) | 4.0  [3.0-6.0]  (0 – 6) | 4.0  [3.0-8.0]  (2 – 8) | 5.0  [4.0-7.0]  (2 – 10) | 6.0  [5.0-8.0]  (3 – 10) |

| The involvement of nurses in providing patient education or information about medication use should be: | Extended | 75 (58.6) | 47 (51.1) | 7 (77.7) | 6 (75) | 16 (38.1) | 12 (60) |
| --- | --- | --- | --- | --- | --- | --- | --- |
|  | Remain the same | 36 (28.1) | 24 (26.1) | 0 | 1 (12.5) | 14 (33.3) | 6 (30) |
|  | Restricted | 0 | 0 | 1 (11.1) | 0 | 2 (4.8) | 0 |
|  | No response | 17 (13.3) | 21 (22.8) | 1 (11.1) | 1 (12.5) | 10 (23.8) | 2 (10) |
| From your current experience, please rate the following items with a score of 0 – 10 (0 = low quality, 10 = high quality) on th quality of…  Median [25^th^ – 75^th^ centile] (full range) | Collaboration between nurses and doctors on providing patient education or information about medication use. | 6.5  [5.0–8.0]  (0–10) | 6.0  [4.0–8.0]  (0–10) | 5.0  [2.0–5.8]  (2–8) | 5.0  [3.0–7.0]  (2 – 7) | 5.0  [4.0–6.0]  (1 – 10) | 5.0  [4.0–7.0]  (2 – 10) |
|  | Collaboration between nurses and pharmacists on providing patient education/information about meds use. | 6.0  [5.0–8.0]  (0–10) | 5.0  [2.0–7.0]  (0–10) | 1.5  [0.0–6.0]  (0–9) | 4.0  [2.0–5.0]  (2 – 5) | 6.0  [5.0–7.0]  (2 – 10) | 5.0  [4.0–7.0]  (2 – 8) |
|  | Nurses’ competence in providing patient education or information about medication use. | 7.0  [6.0–8.0]  (0–10) | 7.0  [5.0–8.0]  (2–10) | 5.0  [2.3–7.0]  (0–7) | 5.0  [2.0–7.0]  (1–7) | 6.0  [4.8–7.0]  (1 – 10) | 6.0  [3.0–7.0]  (2–10) |
|  | Interprofessional communication on providing patient education or information about medication use. | 6.5  [5.0–8.0]  (0 – 10) | 6.0  [4.0–8.0]  (0 – 10) | 5.0  [0.8–5.5]  (0 – 7) | 3.0  [2.0–6.5]  (1 – 8) | 5.0  [5.0–7.0]  (2 – 10) | 6.0  [4.0–7.0]  (2 – 10) |
